# Microbiological quality control for intravaginal devices for the treatment of infectious diseases and bacterial vaginosis

**DOI:** 10.3389/fmicb.2026.1903782

**Published:** 2026-09-10

**Authors:** Christy N. Armstrong, Marnie A. Aagard, Mohamed Y. Mahmoud, Anthony Kyser, Hermann B. Frieboes, Amanda L. Lewis, Warren G. Lewis

**Affiliations:** 1 Department of Obstetrics, Gynecology and Reproductive Sciences, University of California San Diego, La Jolla, CA, United States; 2 Glycobiology Research and Training Center, University of California San Diego, La Jolla, CA, United States; 3 Department of Toxicology and Forensic Medicine, Faculty of Veterinary Medicine, Cairo University, Egypt; 4 Department of Bioengineering, University of Louisville Speed School of Engineering, Louisville, KY, United States; 5 Center for Predictive Medicine, University of Louisville, Louisville, KY, United States; 6 Department of Pharmacology and Toxicology, University of Louisville School of Medicine, Louisville, KY, United States; 7 UofL Health – Brown Cancer Center, University of Louisville, Louisville, KY, United States

**Keywords:** drug delivery, endotoxin, *Lactobacillus*, lipopolysaccharide, quality control, vagina

## Abstract

Preclinical and clinical infectious disease research introducing intravaginal devices containing active agents and probiotic bacteria need to rely on materials that meet appropriate microbiologic standards. However, research methods for quality control of intravaginal devices are poorly described. Here, we develop a practical workflow that specifies media types, culture techniques, and modifications to commonly used assays to suit vaginal applications. A set of quality control assays were developed to test electrospun fibers intended for use in an experimental (mouse) model. The fibers carried probiotics, antibiotics, or no cargo (i.e., “blank”). To validate anti-bacterial action for metronidazole-containing fibers against a relevant target bacterium associated with bacterial vaginosis (BV), a modified Kirby Bauer disc diffusion assay confirmed a zone of *Gardnerella* clearance on NYCIII agar plates. For “blank” fibers containing no cargo, simple turbidity assays using TSB were effective in highlighting batches of electrospun fibers that were contaminated with culturable bacteria. CHROMagar™ Orientation agar was also a useful tool, particularly if fibers contained vaginal probiotic bacteria (*Lactobacillus crispatus*) as the intended cargo. For example, CHROMagar™ identified a probiotic strain other than *L. crispatus* SmR incorporated into fibers in one experiment (*L. rhamnosus* SmR). The limulus amebocyte lysate assay was also used to measure endotoxin levels in specimens from mice and humans, establishing what may be considered “normal” in these settings. Women with an abnormal BV-like microbiota (Nugent score > 4) had ∼250-fold higher levels of endotoxin compared to those without BV (median 134.2 vs. 0.528 EU/ml in vaginal swab elutions). Vaginal washes from uninfected female mice likewise had low concentrations of endotoxin (∼0.6900 EU/ml). Process improvements during the fabrication of electrospinning fibers identified contributing sources of endotoxin and resulted in a 10-fold improvement in median concentrations from 14.21 to 1.143 EU/device. Together, these studies demonstrate a feasible workflow that can quickly identify culturable contaminants or high levels of endotoxin in electrospun fibers intended for vaginal application.

## Introduction

Microbiological quality assurance is an important aspect of product development and FDA regulation. There are extensively documented benefits of quality assurance in the food chain and in drug delivery (Chollet and Jozwiakowski, 2012; Green et al., 2012; Guo et al., 2017; Mahaguna et al., 2004; Morris, 2000; Schleiffer and Speiser, 2022). These quality control procedures protect consumers by reducing exposure to potentially harmful contaminants in medical devices. However, few quality assurance methods are available in the literature directly pertaining to devices for use inside the human vagina or in preclinical models. Previous research in quality assessments of vaginal suppositories has mostly focused on live bacterial contaminants and pH but has failed to look at endotoxin contamination (Mahaguna et al., 2004). Where preclinical model endotoxin research has mostly focused on recalculating the established FDA limits for animal models instead of expanding on endotoxin present in the model itself (Malyala and Singh, 2008). While there has been no set threshold concentration for endotoxin that might be considered healthy in the human vagina, thresholds established by the FDA for mucosal devices are typically set at < 20 EU/device (U.S. Food and Drug Administration, 2012). However, there are few published measurements of endotoxin concentrations in vaginal samples from humans (Aroutcheva et al., 2008; Platz-Christensen et al., 1993), and mice.

Here, our main purpose was to aid early fabrication and preclinical testing efforts by applying microbiological quality testing to ensure high quality research materials are being administered to animal subjects. Additionally, we wanted to determine a relevant endotoxin level for mice and humans for our research material. Quality control methods should ensure antimicrobial activity, flag live contaminants from probable sources, verify intended bacterial content, and ensure contaminants are present below threshold concentrations that could interfere with interpretations related to safety and effectiveness during preclinical stages of research. However, measured concentrations of naturally occurring endotoxins at the site of delivery (the vagina) is also necessary to further inform regulatory science in this active area of research and development. These methods ensure the validity of preclinical research by enabling conclusions that are based solely on the intended active agent(s) delivered.

To establish a framework for quality assurance in the context of our research, we utilized electrospun fibers, which were developed as a potential treatment for bacterial vaginosis (BV). BV is a condition in which the vaginal microbiota maintains lower concentrations of *Lactobacillus* along with a higher abundance and diversity of bacteria and a shift toward a gram-negative, anaerobic population (Ravel et al., 2011; Srinivasan et al., 2013). BV has been associated with many adverse health outcomes, such as preterm birth, sexually transmitted infections, pelvic inflammatory disorders, infertility, urinary tract infection, and cervical cancer (Brotman et al., 2010; Brusselaers et al., 2019; Haggerty et al., 2004; Łaniewski et al., 2018; Ness et al., 2005; Ralph et al., 1999; Spandorfer et al., 2001; Svare et al., 2006; van de Wijgert, 2017; Wiesenfeld et al., 2003). In the U.S., approximately 29% of women met the criteria for BV in a 2007 study (Allsworth and Peipert, 2007), and the estimated annual global cost of symptomatic BV treatment is $4.8 billion (Peebles et al., 2019). The Centers for Disease Control’s (CDC) current recommended treatment regimens are metronidazole (oral or vaginal gel) or clindamycin cream (Reimche et al., 2026). However, alternative treatments range from antimicrobials and antiseptics to acidifying agents and vaginal probiotic bacteria (Tomás et al., 2020).

More than half of those treated for BV will experience recurrences in a matter of weeks or months (Boris et al., 1997; Bradshaw et al., 2006). The high recurrence rates make it challenging to treat the symptoms and prevent adverse outcomes associated with the condition. Therefore, there is a large market for new, evidence-based BV treatments, intravaginal drug delivery methods, and other adjunctive therapies. Many novel devices and treatments have been studied in the past few years with the intent of modifying the vaginal microbiota to create lasting effects that improve health outcomes (Hwang et al., 2020; Kyser et al., 2023; Murina et al., 2018; Utomo et al., 2022; Verstraete et al., 2017).

Electrospun fibers have been one such novel drug delivery method to treat BV. There are many barriers to vaginal drug treatments, such as thick cervical mucus that can fluctuate through hormonal changes, vaginal fluid that contains enzymes that could affect drug stability, wash-out of drugs during the turnover of vaginal secretions, and a lower pH that may affect solubility of drugs. However, electrospun fiber production has many features that aid in drug delivery in the vagina, such as high surface-to-volume ratio, ability to control release of drugs, and high drug-loading efficacy (Sofi et al., 2020). Thus, electrospun fibers have become a useful tool in both probiotic and antibiotic applications. *In vitro* and *in vivo* studies have recently demonstrated that live probiotic lactobacilli can be spun into fibers and release viable bacteria (Armstrong et al., 2025; Mahmoud et al., 2023; Minooei et al., 2023a; Stojanov et al., 2021; Stojanov et al., 2024). Additionally, the antibiotic metronidazole has been incorporated into fibers to show knockdown of Gardnerella, a common bacterium that overgrows in BV (Minooei et al., 2023b). Antibiotics and probiotics have also been shown to exhibit effective release when spun as a single fiber to create a potential dual-treatment approach (Ilomuanya et al., 2023; Verhoeven et al., 2026).

The application of biochemistry, molecular biology, and microbiology approaches are critical for investigating new intravaginal products. Here, practical methods based on FDA guidelines for pharmaceutical microbiology and testing (US FDA) were applied to detect and identify live microbial contaminants or endotoxin in devices meant for intravaginal applications. Specifically, electrospun fibers made of polyethylene oxide (PEO) and poly(lactic-*co*-glycolic acid) (PLGA) have been developed with the intent of delivering antibiotics or probiotic bacteria in preclinical models (Mahmoud et al., 2023; Minooei et al., 2023a,b). These methods can be utilized to assist in the development of safe and effective strategies to disrupt the BV microbiota with antimicrobials and deliver probiotic bacteria such as *Lactobacillus crispatus* to the vagina (Armstrong et al., 2025). We anticipate that these methods can also be used to evaluate other types of intravaginal products.

## Materials and methods

### Electrospun fiber design and fabrication

Briefly, PLGA (50:50, 0.55–0.75 dL/g, 31–57 kDa MW, ≤ 100% PLGA, Lactel Absorbable Polymers Cupertino, CA) and PEO (600,000 MW, ∼80–100% PEG, Sigma Aldrich, St. Louis, MO) were used in the fabrication of the electrospun fibers. PLGA was dissolved in 3 mL of hexafluoro-2-propanol (HFIP, Fisher Scientific, Waltham, MA, USA) at 15% (w/v) and allowed to incubate at 37°C overnight. Likewise, PEO was dissolved in 3 mL of De Man, Rogosa, and Sharpe (MRS) broth at 5% (w/v) and incubated at 37 °C overnight. Incorporation of 5 * 10^7^ colony forming units (CFU) *L. crispatus* was achieved by adding bacteria in 0.5 mL of MRS immediately before electrospinning of a solution containing 150 mg of PEO w/v in 2.5 mL MRS broth. For fibers loaded with metronidazole (MET), the drug was first dissolved in dimethylformamide (DMF) at 15 mg/mL and then 7.5 mg of this solution was mixed into the PEO/MRS solution prior to electrospinning. The mandrel, high voltage power supply, and infusion syringe pumps used to fabricate the electrospun fibers were from Holmarc (Kalamassery, Kochi Kerala, India). During the electrospinning process as described in Armstrong et al. (2025), the ambient temperature was 20°C and the relative humidity was in the range of 15–35%.

Minimizing fiber contamination: To reduce endotoxin levels in polymer solutions, they were passed through a 0.45 μm syringe filter (VWR, PA, USA) after the overnight incubation described above, but before probiotic incorporation. The interior of the box encasing the electrospinner and all materials in contact with fibers were sterilized with ethanol followed by acetic acid prior to electrospinning, and the mandrel was autoclaved for 1 h at 120 °C. Syringe needles were baked for 2 h at 200 °C. Endotoxin free water (Biosciences, MO, USA) and pyrogen-free scintillation vials (VWR, PA, USA) were used.

### Cutting PEO:PLGA electrospun fiber discs

The electrospun fibers were handled within a UV-sterilized biosafety cabinet. Forceps, scissors, and a 1/4-inch arch tool (General^®^ Tools, Secaucus, NJ) were baked at 200 °C for 2 h and autoclaved (121 °C, 15 min.) to prevent LPS or microorganism contamination of the final products. PEO:PLGA fiber discs were cut using a dry-baked and autoclaved 1/4-inch arch tool. Fiber discs were cut to 6 mm diameter, which correlates to approximately 10 mg per disc.

### Bacterial strains

Reference strains of *Lactobacillus crispatus* MV-1A-US, *Escherichia coli* C600, and *L. acidophilus* ATCC4356 were purchased from American Type Culture Collection (ATCC). *Gardnerella* strain JCP8151B was obtained as previously described (Gilbert et al., 2013; Lewis et al., 2013; Robinson et al., 2016).

While attempting to isolate a streptomycin-resistant (SmR) mutant of *L. crispatus*, a spontaneous SmR *L. rhamnosus* was isolated. Sequencing strongly suggested *L. rhamnosus* was chosen for culture and incorporation into fibers instead of *L. crispatus*. This was discovered through our quality control processes, including 16s rRNA PCR and CHROMagar™ Orientation.

### Diffusion sensitivity assay

*Gardnerella* JCP8151B was streaked from a glycerol stock onto anaerobically pre-equilibrated New York City III (NYCIII) agar and incubated at 37°C, anaerobically. Individual colonies were collected and used to make an OD 0.5 suspension in anaerobically pre-equilibrated PBS. A lawn of bacteria was made by dipping a sterile cotton swab into the suspension and swabbing the entire surface of an anaerobically pre-equilibrated NYCIII agar plate. The plate was rotated 90° and the entire surface swabbed again twice more. After the open plate was dried anaerobically at 37°C for 5 min, autoclaved forceps were used to place the 6 mm fiber disc, containing 100 μg MET/mg polymer, onto the plate. Blank fibers that were not loaded with metronidazole were used as a negative control. Assays were performed in triplicate for both blank and metronidazole fibers. Agar plates were incubated anaerobically at 37°C for 24 h before being analyzed. Zones of clearance were measured for each fiber type to determine inhibition of bacterial growth. Anaerobic conditions were achieved using 5% hydrogen gas mix in a vinyl anaerobic chamber (Coy Laboratory Products, Grass Lake, MI) as we have previously described (Morrill et al., 2022).

### Mouse vaginal wash collection and fiber insertion

Mice, obtained from Charles River Laboratories between March and May of 2022, were handled according to institutional guidelines and the Guide for the Care and Use of Laboratory Animals of the University of California San Diego (UCSD, Protocol Number: S20057). Vaginal wash tubes were prepared by pipetting 70 μL of autoclaved Phosphate Buffered Saline (PBS) in a microcentrifuge tube under anaerobic conditions. Mice were manually restrained by gripping the tail and lifting the hindquarters while the mouse was allowed to grasp the top of the cage as previously described (Morrill et al., 2022). 50 μL of PBS were pipetted into the mouse vagina with a P200 pipette. The pipette tip was inserted 2–5 mm into the mouse vagina followed by gentle washing of the vagina by pipetting up and down using the 50 μL setting. Material captured was returned to the original tube still containing 20 μL of anaerobic PBS.

PEO:PLGA fiber discs were inserted into the mouse vagina using sterile metal forceps, as previously described (Armstrong et al., 2025). Metal forceps were baked at 200°C for 2 h and autoclaved at 121 °C for 20 min to ensure sterility and minimize LPS contamination. A sterile P200 pipette tip was used to gently push the disk closer to the cervix.

### Human vaginal specimens

Human vaginal specimens were used for LPS analysis (see below). Supernatants were leftover materials from the Contraceptive CHOICE Project (Secura et al., 2010). Participants of the CHOICE Project gave informed consent at time of enrollment, together with broad permission to use vaginal samples for future studies according to the Institutional Review Board (IRB)- approved protocol at Washington University School of Medicine (IRB ID no. 201108155). However, we note that the current analysis of anonymized samples no longer met the (National Institutes of Health) or University of California definition for human subjects research.

Specimens used in this study were considered based on Nugent score, a microscopy-based scoring method used to determine BV status (Nugent et al., 1991). A score of 0 to 3 corresponds to a sample that contains a predominance of gram-positive rods. In contrast, a score of 7–10 is given for specimens that have few gram-positive rods and instead contain many identifiable bacterial morphotypes that are often gram-negative or gram-variable. A score of 4 to 6 has been referred to as Nugent Intermediate. These specimens often have measurable features similar to the BV condition (Balashov et al., 2014; Cox et al., 2015; Mala et al., 2024; Santos-Greatti et al., 2016). The Nugent scoring method has been extensively described in the literature (Amegashie et al., 2017; Brookheart et al., 2019; Joesoef et al., 1991; Robinson et al., 2019; Tortelli et al., 2020).

### Lipopolysaccharide (LPS) analysis of fibers, murine and human samples

Blank and Probiotic Fiber Samples: One electrospun fiber disc was placed in an Eppendorf tube containing 1 mL of endotoxin-free water (Cytiva, Logan, UT). The tube was heated to 60 °C in an Eppendorf shaker and shaken at 1500 rpm for 30 min. After agitation, the tube was brought to room temperature. Samples were analyzed by the chromogenic Limulus Amoebocyte Lysate (LAL) assay from Cape Cod and Associates (Falmouth, MA). A 10-point standard curve was generated by serially diluting a stock solution 10-fold from 50 EU/mL to 0.005 EU/mL and 15 EU/mL to 0.015 EU/mL. For both standards and samples, 75 μ L were added to a 96-well clear flat-bottom microplate (Greiner Bio-One, Kremsmünster, Austria). The plate was incubated for 10 min at 37 °C, and 75 μ L of LAL reagent were added to each well, and the plate was read for 2 h at 405 nm. The level of detection (LOD) for these assays was 0.0089 EU/disc.

Murine samples: Murine vaginal washes were diluted either 10-fold or 100-fold in endotoxin-free water (Cytiva, Marlborough, MA) before use in the chromogenic Limulus Amoebocyte Assay (LAL) from Cape Cod and Associates.

Human vaginal samples: Swabs were removed from the freezer onto wet ice and eluted by immersing the swabs in 1 mL of buffer (see below) in individual cryo tubes, followed immediately by shaking at 500 rpm for 10 min at room temperature. Cells were separated from the supernatants by centrifugation at 1000 × *g* for 5 min at room temperature. The supernatant was collected and used in the LAL assay. Supernatants were diluted 100-fold in endotoxin-free water before use. Both “normal” and “BV-like” swabs were eluted in either PBS with Roche protease inhibitor or 0.1 M sodium acetate buffer pH 5.5. Buffers were checked for inhibitory properties in the assay (not shown), and neither had an effect on assay read-out. LOD is 0.9 EU/mL.

### Sampling of lab contaminants

Potential microbial sources of lab contaminants were sampled from persons in the Lewis laboratory and were cultured on CHROMagar™ Orientation agar plates. A sterile swab was used to collect samples from the area of interest. For the nasal and oral cavities, the swab was inserted and rolled 3 times on the surface of the cavity. For skin and scalp, the swab was passed over the surface 3 times. Swabs were immediately streaked onto a plate of CHROMagar™ Orientation and incubated in ambient air at 37 °C. The sampling was undertaken solely to identify sources of laboratory contamination, not to generate knowledge about any individual’s health, genetics, or other private attributes. Under UC San Diego’s Human Research Protection Program, a “human subject” is a living individual about whom an investigator obtains identifiable private information or biospecimens for research purposes. In our work the specimens were incidental, not linked to an identifiable person, and the data collected (e.g., viable bacteria from each sample) are about the contaminant, not about the donor. Because the activity does not meet the regulatory definition of human-subjects research, it is exempt from IRB review and no protocol was required.

### Testing for culturable contamination using conventional broth media assay

The broth media turbidity assay was modified from FDA recommendations in the Pharmaceutical Microbiology Manual (U.S. Food and Drug Administration, 2020). Three 6 mm electrospun fiber discs were used for turbidity testing. One disc was used to inoculate 13 mL of Tryptic Soy Broth and incubated in ambient air without shaking at 37 °C for 72 h. Another disc was used to inoculate 13 mL of TSB and incubated in ambient air without shaking at room temperature (∼20 °C) for 120 h. The third disc was used to inoculate 13 mL of Brain Heart Infusion (BHI) Broth and was incubated in an anaerobic chamber at 37 °C for 72 h. The BHI broth was cycled into the anaerobic chamber at least 24 h in advance of inoculation to equilibrate it to anaerobic conditions. After incubation was complete, each solution was assessed for turbidity. Turbidity, as determined visually compared with an uninoculated negative control, indicated a positive test and an elimination of that fiber from use in *in vivo* experiments. A positive control of E. coli C600 was used to confirm turbidity.

### CHROMagar™

Probiotic and blank fibers were both plated on CHROMagar™ Orientation (CHROMagar, Saint-Denis, France) to detect bacterial or fungal contamination. While CHROMagar™ Orientation is typically used to isolate urinary tract pathogens, it also can grow a wide range of other bacteria and fungi, including lactobacilli. Using sterile forceps, a PEO:PLGA disc was placed in a tube containing 1 mL of autoclaved PBS. The tube was vortexed for 15 min to elute the disc. Once maximally dissolved, 250 μ L of the fiber eluent was spread-plated with disposable spreaders (Fisher Scientific, Hampton, NH) on CHROMagar™ Orientation and incubated at 37 °C at 5% CO_2_ for 72 h. Pigmentation of colony growth was analyzed to predict the presence of live contaminants. L. crispatus colonies should be purple. If colonies of different colors were observed, they were individually picked, and PCR sequencing of the 16S rRNA gene was used to determine identity (as described below).

### Isolation of a streptomycin-resistant *Lactobacillus crispatus* MV-1A-US mutant

*L. crispatus* strain MV-1A-US was purchased from ATCC. *L. crispatus* was cultured overnight in MRS broth, anaerobically, at 37°C. The Lactobacillus was plated on pre-equilibrated MRS with 1 mg/mL streptomycin sulfate to select for spontaneous streptomycin-resistant colonies. Both plates and broth were pre-equilibrated to anaerobic conditions. Colonies were cultured in MRS broth. Identity was confirmed by PCR and DNA sequencing (see below).

### Enumeration of *L. crispatus*

For enumeration of probiotic load, fibers composed of PEO fiber were placed in 1 mL PBS and vortexed for 5 min until fully dissolved. PEO fibers were spun with the same inoculum as PEO:PLGA fibers for enumeration purposes, since PEO fibers are fully dissolvable, whereas PEO:PLGA fibers are not. The elution was cycled into the anaerobic chamber and was serially diluted from 10^–1^ to 10^–6^. Dilutions were plated on MRS + 1 mg/mL streptomycin plates that were pre-equilibrated to anaerobic conditions. Plates were incubated at 37 °C for 24 h anaerobically. The resulting colonies were counted and colony forming units (CFU)/mL were calculated using the corresponding dilution factor.

### Strain confirmation of *L. crispatus*

A loaded probiotic fiber was placed in 12 mL of anaerobically pre-equilibrated MRS broth and incubated anaerobically for 16 h at 37 °C. Genomic DNA was extracted from the overnight culture (Monarch Genomic DNA Isolation Kit, New England Biolabs). The 16s rRNA gene was amplified by PCR, using primers FD1 (5’ AGA GTT TGA TCC TGG CTC AG 3’) and RP1/2 (5 ‘ACG GYT ACC TTG TTA CGA CTT 3’). Samples were cycled as follows: 98 °C for 30 s to initiate the process, 28 cycles at 98 °C for 10 s, 46 °C for 30 s, and 72 °C for 90 s, and 72 °C for 10 min to finish the reaction. PCR products were sequenced by EtonBio (San Diego, CA).

### Statistical analysis

Analysis of microbial numbers between days 1 and 7 following administration of fibers was performed using the Mann-Whitney U-test. Likewise, endotoxin levels used descriptive statistics in Graphpad Prism Version 10.4.0. Briefly, Kruskal-Wallis (alpha = 0.05) was used to compare between EF batches and between human vaginal samples with different clinical status (healthy, BV like). The mean ranks were compared between members of preselected groups and Dunns test was used to correct for multiple comparisons. Multiplicity-adjusted P values were reported.

## Results

### Quality control methods for blank, metronidazole-loaded, and lactobacilli-loaded electrospun fibers (PEO:PLGA 3:1)

**FIGURE 1:**
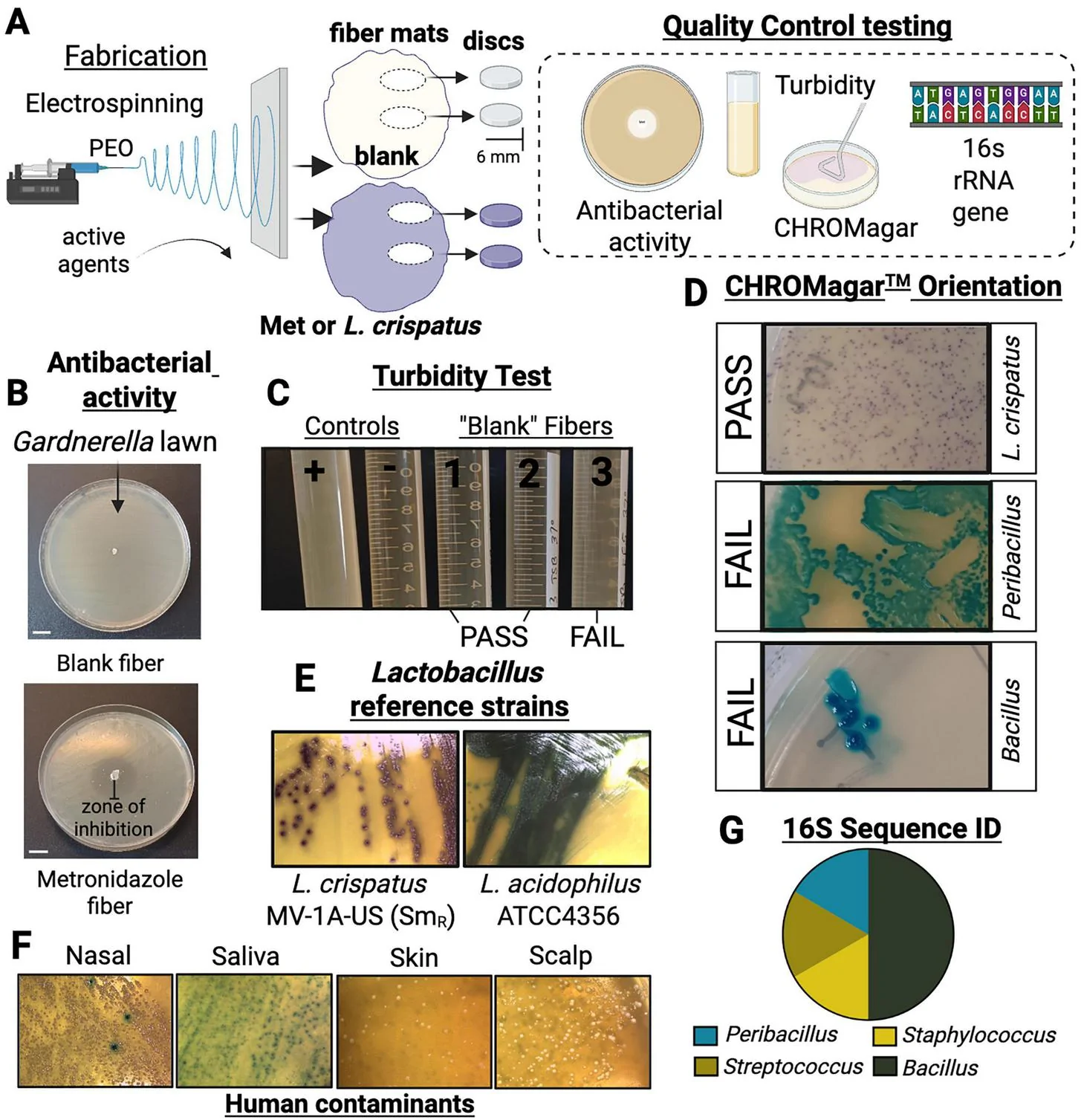
**(A)** Schematic overview of the quality control methods for electrospun fibers fabricated with or without active ingredients. **(B)** Diffusion sensitivity assays using blank- or metronidazole-loaded fibers. **(C)** Turbidity tests and **(D)** CHROMagar™ Orientation were useful in detecting contaminants in electrospun fibers. Culture techniques were validated and used to detect contaminants in fibers without bioactive agents (“blank”), as well as lactobacilli-loaded fibers. **(E)** Reference strains of *L. crispatus* and *L. acidophilus* were directly streaked onto CHROMagar™. **(F)** Human contaminants were used to inoculate CHROMagar™. **(G)** Contaminants found in “blank” fibers using culture-based methods were identified by sequencing the gene encoding 16S RNA.

Quality control methods were developed for metronidazole-loaded, blank, and lactobacilli-loaded fibers (Figure 1A). For metronidazole-loaded fibers, a diffusion sensitivity assay was performed on a lawn of *Gardnerella* (Figure 1B). Blank fibers did not create a zone of inhibition on a lawn of *Gardnerella*. In contrast, fibers loaded with metronidazole (100 μg MET/mg polymer) created an average zone of inhibition radius of 12.3 mm from the scaffold to the edge of the zone.

For blank electrospun fibers, culture-based methods were adapted in parallel to illuminate and identify bacterial contaminants. PEO:PLGA (3:1) fibers were evaluated using a turbidity test in broth and by plating fiber elutions on CHROMagar™ Orientation (Figures 1C, D). CHROMagar™ Orientation was initially validated by using reference strains of lactobacilli and samples of human-derived contaminants (Figures 1E, F) Human-derived contaminants were collected since those were predicted to be one of the most common forms of laboratory contamination.

The turbidity test was assessed for sensitivity by serially diluting *Escherichia coli* strain C600 up to ten times and inoculating Tryptic Soy Broth (TSB) with each dilution. After 24 h of static incubation at 37 °C aerobically, the lowest dilution with turbidity was calculated to be at 1.48 bacterial cells (data not shown). Figure 1C shows an example of turbidity tests that had positive and negative results. The most common bacterial contaminant, as determined by 16s rRNA sequencing, were members of the genus *Bacillus* (Figure 1G). However, *Streptococcus, Staphylococcus*, and *Peribacillus* were also found (Figure 1G).

Eluted fibers, both blank and lactobacilli-loaded, were plated on CHROMagar™ Orientation. Diversity of contamination could be detected by differences in pigmentation. CHROMagar™ was also able to detect laboratory environmental contaminants in probiotic fibers. Figure 1D shows an eluted “blank” fiber with Bacillus contamination, and an eluted probiotic fiber contaminated with Peribacillus.

### CHROMagar™ as a useful tool in quality control of probiotic fibers *in vivo*

**FIGURE 2:**
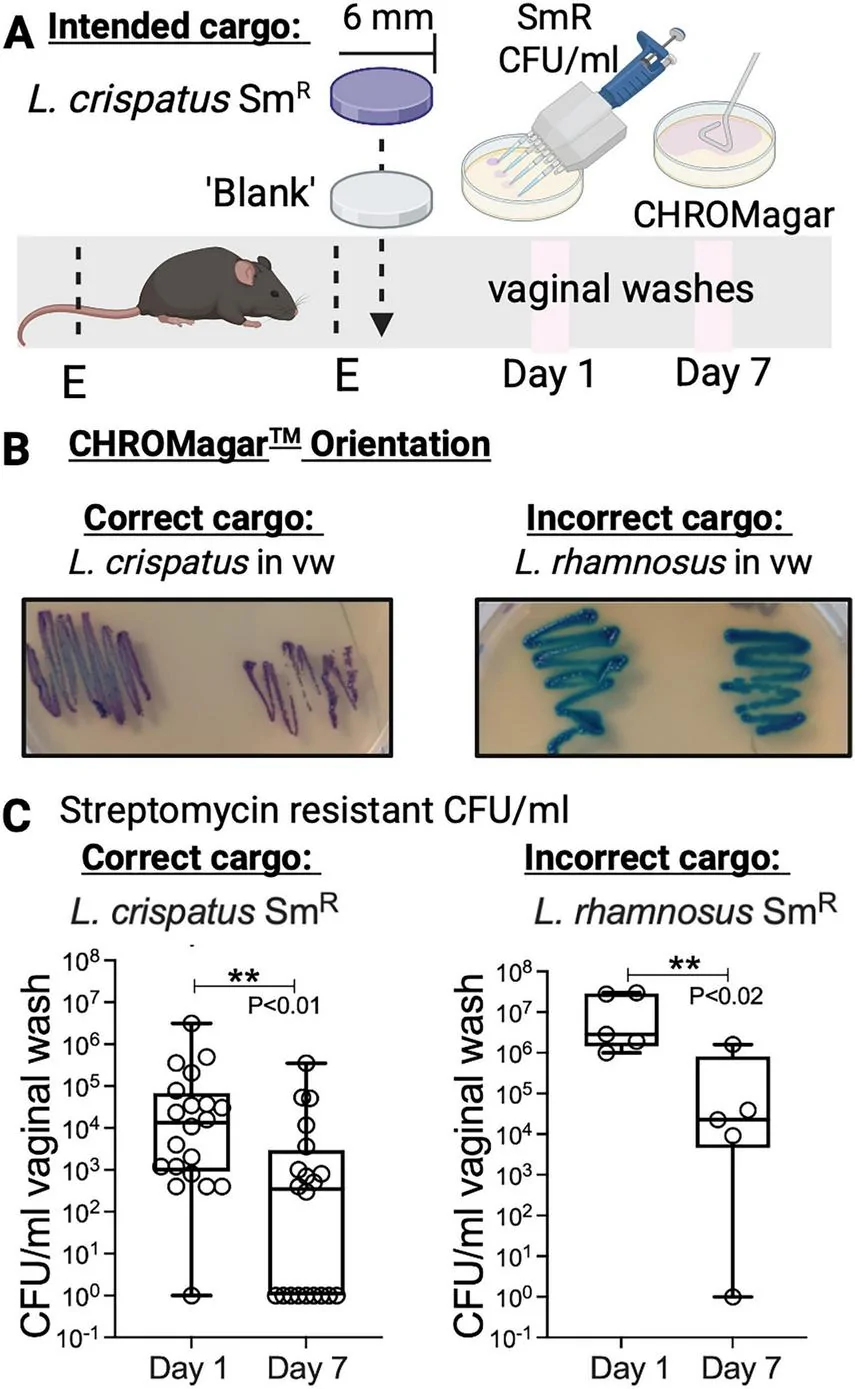
**(A)** CHROMagar™ Orientation able to differentiate probiotic load from contaminants and different *Lactobacillus s*pecies. Even incorrect lactobacilli cargo was utilized in mouse experiments. **(B)** Orientation could also be used to visualize bacterial colonies in mouse vaginal washes. **(C)** Mice were colonized by streptomycin-resistant *L. crispatus* (left) and *L. rhamnosus* (right) after 7 days. The Mann-Whitney U-test was used to compare titers at days 1 and 7 for each microbe showing a statistically meaningful clearance over the studied time period. **P < 0.02.

As previously described, quality control measures were established for PEO:PLGA (3:1) fibers that contain probiotic *Lactobacillus crispatus* (Figure 1). One measure was the use of CHROMagar™ Orientation, which demonstrated that *Lactobacillus crispatus* could be differentiated from other species of *Lactobacillus* (Figure 1E). Thus, fibers loaded with the incorrect lactobacilli could be differentiated not only from 16s rRNA sequencing, but also by culturing methods. Fibers that incorporated the cargo of *L. rhamnosus* were utilized in mouse experiments to test colonization alongside *L. crispatus.* Lactobacilli-loaded fibers were inserted into the vaginas of estrogenized mice. Vaginal washes were performed for 7 days and plated for CFU enumeration (Figure 2A). CHROMagar™ Orientation could be used to differentiate colonies of *L. crispatus* and *L .rhamnosus* from vaginal washes (Figure 2B), and it was found that both species could colonize the mouse vagina after 7 days (Figure 2C). Prior to use in *in vivo* experiments, low LPS concentrations ( < 2 EU/disk) were verified in lactobacilli-loaded fibers using the chromogenic LAL assay (methods previously described).

### Quality control procedures for fabricating electrospun fibers with minimal levels of endotoxin

**FIGURE 3:**
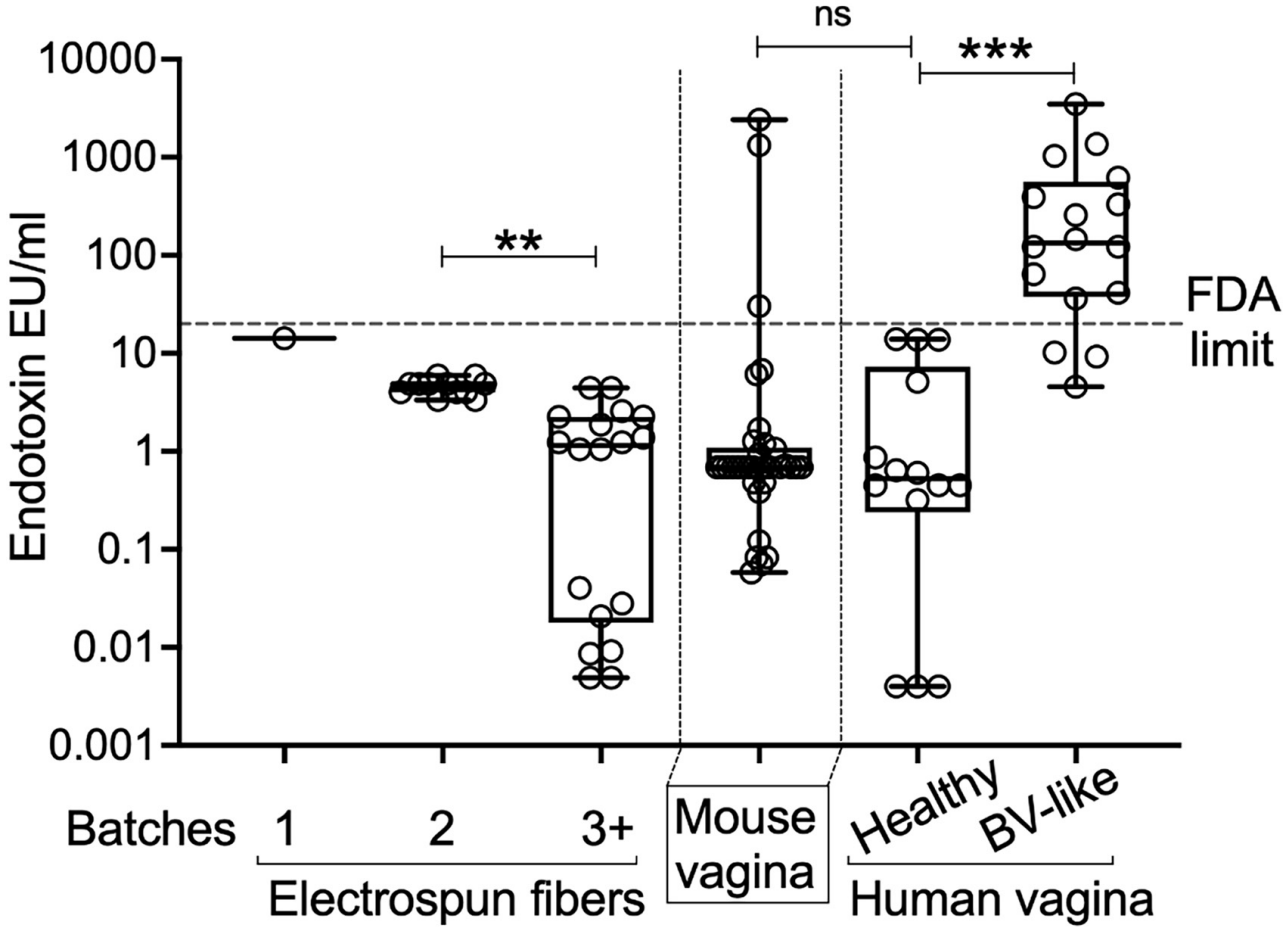
Process improvements to minimize endotoxin in electrospun fiber devices to levels at or below that seen in mouse and human vaginal specimens The chromogenic Limulus Amebocyte Lysate (LAL) assay was used to measure endotoxin/LPS concentrations in different batches of electrospun fibers without active agents. For comparison, LPS concentrations were also measured in mouse vaginal washes, and in human vaginal specimens. Nugent scores of 0–3 correspond to a “No BV” or *Lactobacillus*-dominant status. Nugent score of > 4 indicates an abnormal vaginal microbiota with fewer lactobacilli and the presence of other bacterial morphotypes more indicative of BV (i.e., BV-like). Swabs from each of the experimental groups (No BV and abnormal vaginal microbiota) were eluted in both PBS and sodium acetate buffers. The limit of detection was 0.07 EU/mL and 1.25 EU/mL for two different data sets, reflecting different dilution factors incorporated for individual assays. Kruskal-Wallis with Dunns test was used to examine statistical significance. **P < 0.02; ***P < 0.0001.

Electrospun fibers were first tested without the addition of bioactive products (blank fibers, Figure 1). The initial batch of fibers had endotoxin levels of 12.21 Endotoxin Units (EU) (Figure 3) Reducing endotoxin contamination was achieved by switching to endotoxin-free water, dry-baking the syringe needles, switching to pyrogen-free plastic materials, and by sterilizing the electrospinning box and all materials in contact with the fibers. Endotoxin levels ranged from 3.345–5.969 EU/disc in the second batch of fibers. The levels were further reduced by replacing older syringe units on the electrospinning box, resulting in the next 3 batches having median endotoxin levels of 1.143 EU/disc (Figure 3).

Endotoxin levels were also measured in mouse vaginal washes and eluted materials from human vaginal swabs for comparison. Endotoxin concentrations in mouse vagina were low overall, but variable, with a median value of 0.6900 EU/mL (95% CI:0.6900–0.7218) (Figure 3). There was no clear correlation between endotoxin levels and the stage of the estrus cycle (not shown).

Next, leftover material from human vaginal swab elutions with known BV status (Agarwal et al., 2023; Secura et al., 2010) were tested. Swabs were eluted in buffer, and cells and supernatant were separated. Supernatants from these elutions were assessed for their free endotoxin concentration using the LAL assay. Nugent scores indicate the microbiological status of the vagina. Specimens without BV, as determined by a Nugent score of 0–3, had low median endotoxin concentrations (median 0.5280 EU/mL; 95% CI: 0.0040–13.78 EU/mL). Specimens with abnormal vaginal flora, as determined by a Nugent score of > 4 held more than 100-fold higher endotoxin concentrations (median 134.2 EU/mL; 95% CI: 36.32–621.3 EU/mL) (Figure 3).

## Discussion

The quality assurance methodology in this paper was developed and modified from the guidelines from the FDA (U.S. Food and Drug Administration, 2020). For sterility testing, the FDA recommends incubating the device in Soybean-Casein Digest (another term for TSB), aerobically, at 22.5 ± 2.5 °C. In parallel, they recommend incubating the device in Fluid Thioglycollate media, anaerobically, at 32.5 ± 2.5 °C. Furthermore, the FDA recommends incubating devices for no less than 14 days in broth (U.S. Food and Drug Administration, 2020). Changes to incubation conditions were made to enable quality assurance to be expedited for each batch of fibers to maximize viability of probiotic *Lactobacillus* prior to fiber introduction into preclinical models. Brain-Heart Infusion broth replaced Fluid Thioglycollate Media (FTM) to reduce cost and allow easier analysis of more batches and samples. With the use of an anaerobic chamber, FTM’s oxygen-binding capabilities and oxygen gradient would not be necessary nor functional. In addition, CHROMAgar™ Orientation was incorporated as a way to differentiate colonies that may look similar using other media types. This incorporation reflects the FDA recommendations for testing non-sterile products, which suggest utilizing molecular methods or general enrichment plates to screen test samples for microorganisms that are not intended to be in the device (U.S. Food and Drug Administration, 2020). CHROMAgar™ Orientation was also effective at differentiating lactobacilli from mouse vaginal washes. This could assist researchers in analyzing *in vivo* colonization of devices with multiple species of lactobacilli.

Additionally, the Kirby-Bauer diffusion sensitivity test was modified to validate antimicrobial activity of antibiotic-loaded fibers. Application of the Kirby-Bauer disc diffusion assay is one of the key methods used to evaluate the effectiveness of antibiotics released from paper discs on target bacterial pathogens (U.S. Food and Drug Administration, 2020). In this assay, the paper discs are placed on top of agar plates that are pre-inoculated to generate a lawn of potentially susceptible bacteria. The method was here adapted to test the capability of electrospun fiber discs to release metronidazole, a common antibiotic used to treat BV. Further, the method was adapted to anaerobic conditions to specifically test the sensitivity of *Gardnerella*, one of the most highly enriched bacterial taxa in BV, to metronidazole released from the fiber. Methodology was adapted to use NYCIII agar in lieu of Mueller-Hinton agar to promote the growth of *Gardnerella.*

Our analysis of endotoxin (LPS) concentrations in mouse and human vaginal specimens provides critical context for FDA regulatory limits. Healthy human vaginal samples (Nugent score 0–3) exhibited median endotoxin levels of ∼1.5 EU/mL, while BV-associated samples exceeded 100 EU/mL, reaching up to 935 EU/mL. Mouse vaginal washes showed low levels (∼0.75 EU/mL). In contrast, FDA guidance (USP <85>/<161> ) sets a conservative limit of ≤ 20 EU/device for mucosal-contact products (U.S. Food and Drug Administration, 2012). This threshold is well below physiological levels in healthy vaginal environments and far below BV-associated dysbiosis, ensuring that devices do not introduce clinically significant endotoxin burdens. Our fiber devices achieved < 2 EU/disc after process improvements, aligning with FDA standards and supporting their safety for vaginal application. Thus, the FDA requirement appears reasonable and protective, providing a robust safety margin relative to natural conditions and pathological states. Additionally, endotoxin testing can be utilized for blank, probiotic, and antibiotic fibers. The probiotic used, *L. crispatus*, is a Gram-positive bacterium and does not have LPS, so the chromogenic LAL assay can be used to detect additional LPS. Metronidazole did not appear to affect the assay either.

A limitation of the quality assurance methodology is that although it was able to provide process improvements to lower endotoxin levels in the fibers, it was not able to reduce the number of positive turbidity tests through our processes. While it was able to detect culturable contaminants, the current methodology is limited to only detection and not prevention. Additionally, potential bacterial toxins other than LPS that may elicit an immune reaction were not tested. More testing and research is needed to establish the framework to assess the presence of toxins that may be contaminants in the absence of culturable, live microorganisms.

Quality assurance is a necessary step in validating new devices, however bacteria-loaded devices can pose a particular challenge. The processes and data shown here establish a quality control workflow for intravaginal devices that can detect and identify potential contaminants, such as LPS or live microbes. These processes also guarantee fiber load, such as probiotics or antimicrobials. The results demonstrate that with simple process improvements, endotoxin levels can be reduced in devices to well below the recommendations of the FDA. This research also provided endotoxin concentrations for the mouse and human vaginal fluid for comparison. Finally, the methods can be expanded for use in preclinical experiments by analyzing probiotics from mouse vaginal washes. This research does not aim to change the FDA or US pharmacopoeia regulations, nor is it intended to replace medical device sterility standards. It aims to provide a faster, less expensive, more specific framework for researchers who wish to incorporate quality control measures for their preclinical intravaginal devices. These measures are necessary for the advancement of future devices to treat BV and represent useful tools for preclinical studies focusing on the female reproductive tract.

## Data Availability

The raw data supporting the conclusions of this article will be made available by the authors, without undue reservation.
